# Atypical Presentation of Idiopathic Bilateral Optic Perineuritis in a Young Patient

**DOI:** 10.1155/2016/6741925

**Published:** 2016-12-18

**Authors:** Jessica Mani Penny Tevaraj, Evelyn Tai Li Min, Raja-Azmi Mohd-Noor, Lakana Kumar Thavaratnam, Win Mar Salmah, Wan Hazabbah Wan Hitam

**Affiliations:** ^1^Department of Ophthalmology, School of Medical Sciences, Universiti Sains Malaysia, Health Campus, 16150 Kubang Kerian, Kelantan, Malaysia; ^2^Hospital Universiti Sains Malaysia, 16150 Kubang Kerian, Kelantan, Malaysia; ^3^Department of Radiology, School of Medical Sciences, Universiti Sains Malaysia, Health Campus, 16150 Kubang Kerian, Kelantan, Malaysia

## Abstract

A previously healthy 27-year-old Malay male presented with acute onset of painless, severe blurring of vision in his right eye. It was associated with headache and vomiting for the past week. Relative afferent pupillary defect was present in the right eye, with reduced optic nerve function. Patient also had bilateral generalised optic disc swelling, splinter haemorrhages, and tortuous vessels. Initial examination was suggestive of either optic neuritis or raised intracranial pressure. Typical features of bilateral optic perineuritis (OPN) such as tram track and doughnut sign were observed on magnetic resonance imaging. Connective tissue and infective screening were negative. He was diagnosed with bilateral optic perineuritis and treated with high dose intravenous corticosteroids followed by a three-month course of oral steroids. His vision and optic nerve function recovered to baseline levels.

## 1. Introduction

First described in 1883, optic perineuritis is an uncommon orbital inflammatory disease involving the optic nerve sheath; although it tends to mimic demyelinating optic neuritis, there are some clinical features that help to distinguish the two [1]. It is often mistaken for optic neuritis, resulting in inappropriate treatment and frequent relapses. Optic perineuritis typically presents with acute unilateral optic nerve dysfunction and pain exacerbated by eye movement [1]. We report an atypical presentation of painless bilateral optic perineuritis in a young adult with initial symptoms mimicking that of increased intracranial pressure.

## 2. Case Report

A previously healthy 27-year-old Malay male presented with acute, severe blurring of vision of the right eye for one week, associated with frontal headache and vomiting. There was no pain on eye movement and no other history suggestive of infective or connective tissue disease.

**Figure 1:**
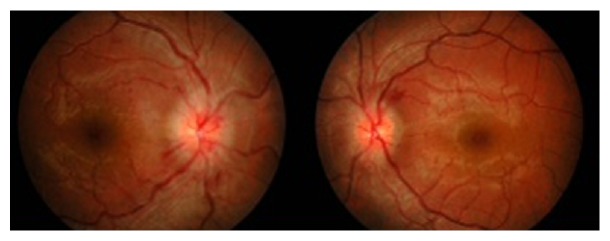
Fundus photo on presentation showing bilateral generalised optic disc swelling with splinter haemorrhages.

The visual acuity of the right eye was counting fingers at 2 feet, while that of the left eye was 6/18, improving to 6/12 with pinhole. The relative afferent pupillary defect was positive in the right eye. Red saturation and light brightness were reduced in the right eye, with absence of colour vision. Confrontation test showed a central scotoma in the right eye, which extended superiorly to involve the paracentral area. The left eye initially had normal optic nerve function tests and visual field, but the colour vision deteriorated two days after his initial presentation. The anterior segment findings of both eyes were normal. Posterior segment examination revealed bilateral generalised optic disc swelling, tortuous vessels, and splinter haemorrhages, which were more in the right eye (Figure 1). Both eyes had a normal macula. Except for the optic nerve, the remainder of the neurological examination was normal. The differential diagnoses at this point included raised intracranial pressure, bilateral optic neuritis, and meningeal infiltration.

An urgent computed tomography scan showed bilateral enhancement of the optic nerves suggestive of optic neuritis, with normal brain parenchyma. Magnetic resonance imaging (MRI) demonstrated abnormal enhancement surrounding the intraorbital optic nerves, seen as characteristic “doughnut sign” on coronal views and “tram track” sign on axial views. Slight tortuosity of the optic nerve was seen on the right side. Connective tissue and infective screening including venereal disease research laboratory test (VDRL), angiotensin converting enzyme, antineutrophil cytoplasmic antibodies, antinuclear antibodies, retroviral serology, chest X-ray, and Mantoux test were normal.

**Figure 2:**
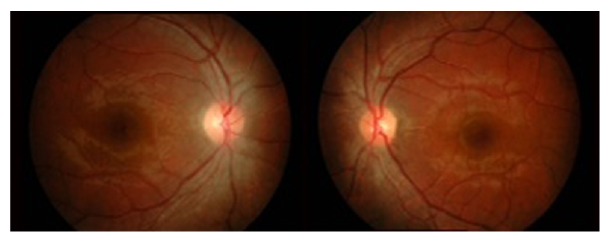
Fundus photo after three weeks of treatment showing regression of the optic disc swelling and splinter haemorrhages.

Intravenous methylprednisolone 250 mg QID was commenced and continued for three days (a total of 12 doses), after which the patient was prescribed oral prednisolone 1 mg/kg/day, which was tapered slowly over the following 3 months. Three weeks posttreatment, the best corrected vision improved to 6/21 on the right eye and 6/7.5 on left eye, with normal pupillary reaction. However, colour vision remained poor bilaterally (1/15 plates). There was regression of the optic disc swelling and splinter haemorrhages (Figure 2).

**Figure 3:**
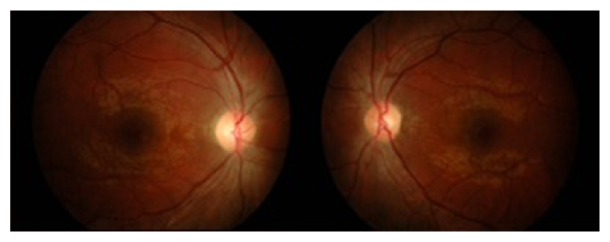
Fundus photo after six weeks of treatment showing resolution of the optic disc swelling and haemorrhages bilaterally.

After six weeks of prolonged steroids on tapering dose, the vision improved to 6/6 in both eyes, with normalized optic nerve function tests including colour vision (15/15 plates) and resolution of the optic disc swelling and haemorrhages bilaterally (Figure 3). The patient remains relapse-free one year later.

## 3. Discussion

Idiopathic orbital inflammatory diseases, sometimes referred to as orbital pseudotumours, have a wide spectrum of clinical manifestations, ranging from focal to diffuse involvement of the periocular structures [2]. These include myositis, dacryoadenitis, scleritis, and perineuritis, all of which appear to be more common in women [3]. Most cases of optic perineuritis are isolated or idiopathic, but associations with Wegener's granulomatosis, giant cell arteritis, sarcoidosis, tuberculosis, and syphilis have been documented [1, 2, 4, 5].

Unlike optic neuritis, which mainly involves a younger age group, optic perineuritis can be manifested in a broad age range [1]. Delayed presentation is more common in the latter as the onset is often subacute, followed by a progressive decrease of vision [1]. In a typical presentation of optic perineuritis, the patient complains of unilateral blurring of vision associated with retroorbital pain and pain on eye movement [1]. Bilateral presentation is rare: in a review of 14 cases which presented to two different centres, only one case was bilateral [1]. Although the visual field involvement may be variable [6], optic perineuritis tends to spare the central vision [1]. Our patient was atypical in that he had acute, painless, and severe bilateral involvement, with a right eye central scotoma. Also, the coexistence of optic disc swelling with headache and vomiting in this patient mimicked an intracranial pathology rather than an orbital pathology [1].

**Figure 4:**
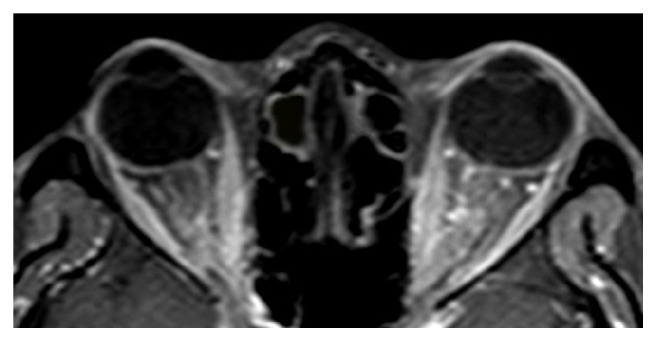
Contrast-enhanced MR images showing marked enhancement of the optic nerve sheath on axial view (tram track sign). Slight tortuosity of the optic nerve is seen on the right side.

**Figure 5:**
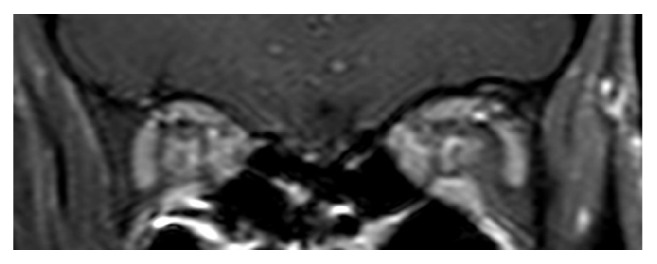
Contrast-enhanced MR images showing marked enhancement of the optic nerve sheath on coronal view (doughnut sign).

As the differential diagnoses of bilateral OPN include intracranial pathology with raised intracranial pressure and optic neuritis, MRI plays an invaluable role in differentiating between these disorders and OPN. Typical MRI features of OPN are the “tram track” sign of the optic nerve sheath on axial views (Figure 4) and the “doughnut sign” on coronal views [1] (Figure 5). These features are better seen in MRI fat suppression and postgadolinium orbital views, compared to a basic CT scan [1]. In cases of atypical presentation of bilateral OPN, MRI is especially useful as a diagnostic aid and can obviate the need for further invasive investigations such as lumbar puncture.

Although optic perineuritis and optic neuritis have similar presentations, the treatment differs in two aspects. First, commencing high dose oral corticosteroids alone has been observed to increase the risk of recurrence in optic neuritis but not optic perineuritis [3, 7]. Secondly, in optic neuritis, oral prednisolone is given for eleven days and then is stopped; however in optic perineuritis, there is a need for a longer duration of oral corticosteroids. In our case, high dose intravenous corticosteroids were given for 3 days, followed by a slow tapering dose of oral prednisolone for 6–8 weeks [8]. The prolonged duration of corticosteroids is to reduce the risk of relapse, as repeated insults to the optic nerve may result in irreversible damage.

## 4. Conclusion

Although OPN is generally characterized by unilateral, painful, optic nerve dysfunction, the spectrum of its clinical presentation is wide. The clinician should have a high index of suspicion for this condition because rarely it may manifest as bilateral, painless optic neuropathy with significant visual impairment. As bilateral OPN may mimic a spectrum of diseases, MRI is a useful part of the diagnostic workup.
